# Frequency of Subclinical Hypothyroidism Among Pregnant Women: A Retrospective Chart Review

**DOI:** 10.7759/cureus.41629

**Published:** 2023-07-10

**Authors:** Aliya Islam, Shaharyar K Niazi

**Affiliations:** 1 Public Health, SurgiMed Hospital, Islamabad, PAK

**Keywords:** thyroid disorders, retrospective chart review, thyroid hormone profile, pregnancy, subclinical hypothyroidism

## Abstract

Background

Subclinical hypothyroidism is a common endocrine disorder during pregnancy, associated with adverse maternal and fetal outcomes. This study aimed to evaluate the frequency of subclinical hypothyroidism among pregnant women who presented to our hospital between 2020 and 2022.

Methods

A retrospective chart review was conducted on 589 pregnant women using convenience sampling. Only women who underwent thyroid function testing and had no known thyroid disease were included. Data on age, weight status, history of miscarriage, history of infertility, menstrual cycle regularity, thyroid function, and symptoms of thyroid diseases were collected. Descriptive statistics were used to analyze the data.

Results

The mean age of the participants was 24.8 ± 3.7 years. Among the participants, 270 (45.9%) had a normal weight, 199 (33.8%) were overweight, and 120 (20.4%) were obese. A history of miscarriage was reported by 69 women (11.7%) while 37 women (6.3%) had a history of infertility. The menstrual cycle was regular in 499 women (84.7%) and irregular in 90 women (15.3%). The results showed that 517 (87.7%) women were euthyroid, 47 (7.9%) had hypothyroidism, and 25 (4.2%) had hyperthyroidism. Of the 47 patients with hypothyroidism, 32 (68.08%) had subclinical hypothyroidism and 15 (31.91%) had overt hypothyroidism.

Conclusion

This study highlights the frequency of subclinical hypothyroidism among pregnant women. The findings underscore the importance of thyroid function testing during pregnancy.

## Introduction

Subclinical hypothyroidism is a common endocrine disorder characterized by elevated serum thyroid-stimulating hormone (TSH) levels and normal free thyroxine (fT4) levels. It is a condition that falls within the spectrum of thyroid dysfunction, ranging from overt hypothyroidism to subclinical hypothyroidism to euthyroidism. Overt hypothyroidism is characterized by both elevated TSH levels and decreased T4 levels while subclinical hypothyroidism is defined by elevated TSH levels with normal fT4 levels [1].

During pregnancy, the thyroid gland undergoes significant changes to meet the increased demands for thyroid hormone production. Thyroid hormones play a critical role in fetal neurodevelopment, growth, and metabolism. Therefore, any disruption in thyroid function during pregnancy can have significant implications for both maternal and fetal health [2].

Subclinical hypothyroidism during pregnancy has been associated with adverse outcomes, including gestational hypertension, preeclampsia, preterm birth, low birth weight, and cognitive impairment in offspring [3]. The exact mechanisms underlying these associations are not fully understood but are believed to be related to the disruption of normal thyroid hormone signaling and the resulting effects on placental function, fetal development, and maternal vascular health.

Despite its clinical significance, the frequency and profile of subclinical hypothyroidism among pregnant women are not yet well understood, particularly in specific healthcare settings. The prevalence of subclinical hypothyroidism during pregnancy varies widely in different populations and geographical regions, ranging from 2% to 18% [3,4]. This wide variation may be attributed to differences in study design, diagnostic criteria, population characteristics, and geographic factors.

This study aimed to evaluate the frequency of subclinical hypothyroidism among pregnant women. A retrospective chart review was conducted to assess the thyroid hormone profile and symptoms related to thyroid diseases in this population. By examining the frequency and profile of subclinical hypothyroidism in this specific healthcare setting, we aim to contribute to the existing literature and provide valuable insights for clinical practice.

## Materials and methods

Study design

This retrospective chart review aimed to evaluate the frequency and profile of subclinical hypothyroidism among pregnant women who presented to SurgiMed International Hospital, a tertiary-care hospital, between 2020 and 2022. The need for patient consent was waived due to the retrospective nature of the study.

Participants

Convenience sampling was employed to include 589 pregnant women who underwent thyroid function testing as part of routine antenatal care. Our hospital received an estimated 2895 new pregnant women during the same time; therefore, 20.3% of the total pregnant women were included in the study. Women underwent thyroid testing at the first visit at the discretion of the treating doctor. In our hospital laboratory, the normal fT4 level was 0.81-1.42 ng/dL, the normal TSH level was 2.5-4.0 mIU/L, and the normal fT3 level was 60-180 ng/dL. Subclinical hypothyroidism was defined as TSH level > 4.0 mIU/L and normal fT4 level, whereas overt hypothyroidism was defined as TSH level > 4.0 mIU/L and fT4 level < 0.81 ng/dL. Women with known thyroid diseases were excluded from the study to ensure a more accurate representation of subclinical hypothyroidism prevalence among previously undiagnosed cases.

Data collection

Data on various variables were collected from electronic medical records. These variables included age, weight status, dyslipidemia, history of miscarriage, history of infertility, menstrual cycle regularity (as a possible indicator of previous thyroid disease), and thyroid function. Weight status was determined based on body mass index (BMI) criteria, classifying women as normal weight (18.5-24.9 kg/m^2^), overweight (25.0-29.9 kg/m^2^), or obese (> 30 kg/m^2^) [5].

Statistical analysis

Descriptive statistics, such as mean, standard deviation, and percentages, were used to summarize the data collected. The prevalence of subclinical hypothyroidism and other variables of interest were calculated to provide insights into the profile of pregnant women with subclinical hypothyroidism in the study population.

## Results

The mean age of the participants was 24.8 years (SD: 3.7). The mean gestational age was 15 ± 8.9 weeks at the time of laboratory tests. Among the 589 pregnant women included in the study, 270 (45.9%) had a normal weight, 199 (33.8%) were overweight, and 120 (20.4%) were obese. Additionally, a history of miscarriage was reported by 69 women (11.7%), and 37 women (6.3%) had a history of infertility. The menstrual cycle was regular in 499 women (84.7%) while 90 women (15.3%) reported irregular cycles. Of the 90 women with irregular cycles, 60 (66.67%), 21 (35%), and nine (10%) had euthyroidism, hypothyroidism, and hyperthyroidism, respectively. Of the 21 women with irregular cycles and hypothyroidism, 14 (66.67%) and seven (33.33%) had subclinical and overt hypothyroidism, respectively. Further details regarding the demographic and clinical characteristics are presented in Table 1.

**Table 1:** Demographic and clinical characteristics of study participants

Variable			
		Mean	Standard Deviation
Age		24.8 years	3.7
Gestational age		15 weeks	8.9
		Number	Percentage
Weight	Normal weight	270	45.9%
	Overweight	199	33.8%
	Obese	120	20.4%
History of miscarriage		69	11.7%
History of infertility		37	6.3%
Menstrual irregularity		90	15.3%

The results of thyroid function testing revealed that 517 (87.7%) women were euthyroid, 47 (7.9%) had hypothyroidism, and 25 (4.2%) had hyperthyroidism (Table 2). Of the 47 patients with hypothyroidism, 32 (68.08%) had subclinical hypothyroidism and 15 (31.91%) had overt hypothyroidism.

**Table 2:** Frequency of thyroid disorders

Variable	Number	Percentage
Euthyroidism	517	87.7%
Hypothyroidism	47	7.9%
Hyperthyroidism	25	4.2%

## Discussion

The findings of this retrospective chart review demonstrate a significantly high frequency of subclinical hypothyroidism among pregnant women. Previous studies have reported an association between subclinical hypothyroidism and adverse pregnancy outcomes [6]. The identification and appropriate management of subclinical hypothyroidism during pregnancy are crucial for optimizing maternal and fetal health [7].

Subclinical hypothyroidism during pregnancy can lead to hormonal imbalances that may disrupt the normal physiological processes essential for successful gestation [8,9]. Furthermore, the association of subclinical hypothyroidism with a history of miscarriage and infertility highlights the need for early detection and intervention in women planning to conceive or those who are already pregnant [10].

Regular menstrual cycles were more prevalent among the study participants, which may indicate a healthier endocrine environment and a lower risk of subclinical hypothyroidism. However, the presence of subclinical hypothyroidism in a considerable proportion of women with regular menstrual cycles emphasizes the importance of routine thyroid function testing during pregnancy, even in apparently healthy individuals.

Despite the valuable insights provided by this study, several limitations should be considered. First, the study design was retrospective and relied on the data available in electronic medical records. This introduces the possibility of incomplete or missing information, as well as potential inaccuracies in the documentation. Additionally, the use of convenience sampling may limit the generalizability of the findings to the broader population of pregnant women. A larger, prospective study with a more diverse sample would help validate and generalize the results.

Another limitation is the lack of information regarding the etiology and duration of subclinical hypothyroidism. Understanding the underlying causes and the duration of the condition could provide valuable insights into its progression and potential implications for pregnancy outcomes. Future studies should aim to collect such data to enhance our understanding of subclinical hypothyroidism during pregnancy.

Furthermore, this study focused on the frequency and profile of subclinical hypothyroidism but did not explore the specific adverse outcomes associated with the condition in this cohort. Future research should investigate the impact of subclinical hypothyroidism on maternal and fetal health outcomes, including gestational hypertension, preterm birth, and cognitive development in offspring. Long-term follow-up studies are needed to assess the potential effects of subclinical hypothyroidism on the health and well-being of both mothers and children.

## Conclusions

This retrospective chart review reveals a significant frequency of subclinical hypothyroidism among pregnant women. The findings underscore the importance of routine thyroid function testing during pregnancy. Future prospective studies are warranted to further explore the implications of subclinical hypothyroidism and its association with adverse pregnancy outcomes, allowing for the development of evidence-based guidelines for screening, diagnosis, and management.
